# Comparison of effects of Empagliflozin and Linagliptin on renal function and glycaemic control: a double-blind, randomized clinical trial

**DOI:** 10.1186/s40842-022-00142-1

**Published:** 2022-05-25

**Authors:** Mohammad Amin Mohammad zadeh Gharabaghi, Mohammad Reza Rezvanfar, Nasser Saeedi, Faezeh Aghajani, Mohammad Alirezaei, Pourya Yarahmadi, Amin Nakhostin-Ansari

**Affiliations:** 1 Internal Medicine Department, Arak University of Medical Sciences, A’lam-Al-Hoda Street, Shahid Shiroodi Street, Arak, Iran; 2 Research Development Center, Arash Women’s Hospital, Tehran University of Medical Sciences, Tehran, Iran; 3 School of Medicine, Tehran University of Medical Sciences, Tehran, Iran; 4 Sports Medicine Research Center, Neuroscience Institute, Tehran University of Medical Sciences, Tehran, Iran

**Keywords:** Albuminuria, Diabetes, Empagliflozin, Linagliptin

## Abstract

**Background:**

This study aimed to compare the effects of Linagliptin and Empagliflozin on renal function and glycaemic control in patients with type 2 diabetes mellitus (DM).

**Method:**

We conducted a randomized, double-blind, parallel trial on patients aged 30 to 80 years with type 2 DM and HbA1c ≤ 9%, regardless of background medical therapy, to compare the effects of Empagliflozin and Linagliptin on albuminuria, FBS, HbA1c, and eGFR. Participants were given the mentioned drugs for 12 weeks. Statistical analysis was performed using appropriate tests in IBM™SPSS® statistics software for windows version 24.

**Results:**

In total, 60 patients participated in the study, thirty patients in each group. The mean age of participants was 56.8 (SD = 8.15) in the Empagliflozin group and 60.9 (SD = 7.22) in the Linagliptin group. Before the intervention, FBS, HbA1C, and albuminuria values were significantly higher in the Empagliflozin group than those in the Linagliptin group (*P* < 0.05), but there was no significant difference between groups regarding eGFR (*P* = 0.271). Changes in the FBS, HbA1C, and eGFR were not significantly different between groups (*P* > 0.05), but there was more decrease in albuminuria in the Empagliflozin group compared to the Linagliptin group (*P* = 0.001, Cohen’s d = 0.98).

**Conclusions:**

Regardless of baseline albuminuria, eGFR, or HbA1c, Empagliflozin 10 mg daily significantly reduced albuminuria at 12 weeks compared to Linagliptin 5 mg daily in patients with type 2 diabetes.

**Trial registration:**

Iranian Registry of Clinical Trials, IRCT20200722048176N1. Registered 3 August 2020.

## Background

Diabetes Mellitus (DM) is a debilitating metabolic disorder characterized by impaired insulin function, leading to chronic hyperglycemia [1]. Two main types of DM are type 1, insulin-dependent, and type 2, insulin-independent, and insulin resistance plays a crucial role in type 2 DM [2]. In 2017, it was estimated that the global prevalence of type 2 DM is about 6.28%, with about 462 million people being affected [3]. Additionally, approximately 193 million diabetic patients worldwide remain undiagnosed, putting them at risk of untreated chronic hyperglycemia complications [4]. Chronic hyperglycemia can cause microvascular and macrovascular complications. Several metabolic and structural alterations contribute to these vascular complications, such as the accumulation of glycation end products, improper stimulation of signal pathways like protein kinase C and hemodynamic regulatory mechanism of the renin–angiotensin–aldosterone system (RAAS), and excessive production of reactive oxygen species (ROS) [5].

Diabetic nephropathy (DN) is one of the most common and severe microvascular complications of DM, occurring in about 20–30% of diabetic patients [6, 7], which is typically defined as increased excretion of protein in the urine [8]. However, in some countries, as high as 45% of patients with type 2 DM are reported to have DN [9]. One of the major consequences of DN is kidney failure, leading to end-stage renal disease (ESRD), advanced cardiovascular disease, and death [10]. Even with extensive lifestyle and drug interventions [11], DN still accounts for the majority of cases of ESRD and triple the risk of dying from it [12].

A growing concern during the course of type 2 DM is the prevention or delay of the progression of DN, especially with its increasing incidence each year. Early-stage DN causes glomerulosclerosis, compensatory hypertrophy, and late-stage DN leads to gradual atrophy [13]. Treatment of DN consists of different interventions, including changes in lifestyle, glycaemic control, and pharmaceutical treatments [14]. Poor glycaemic control is associated with more severe DN [15], with glycaemic control can prevent the incidence of DN and slow its progression [16]. Glycemic control can also, in the long term, reverse some kidney histological changes in patients with type 2 DM [17]. Therefore, glycaemic control is one of the cornerstones of type 2 DM treatment [14]. Sodium-glucose cotransporter-2 inhibitors (SGLT2i) are one of the suggested pharmaceutical agents for glycaemic control in patients with diabetes, and concurrent DN, especially those with an estimated glomerular filtration rate (eGFR) of higher than 30 ml/min, high risk of hospitalization due to heart failure or atherosclerotic cardiovascular diseases [18]. In addition to glycaemic control, SGLT2 inhibitors can prevent glomerular injury. There are some hypotheses regarding the mechanisms of these beneficial effects of SGLT2 inhibitors on renal function, including their positive effects on risk factors of DN, reducing glomerular capillary pressure, decreasing inflammation, activating renin-angiotensin system (RAS), and decreasing podocyte damage; however, the exact mechanism is unknown [19]. Empagliflozin is an SGLT2i that has anti-fibrotic and anti-inflammatory effects and can slow the progression of DN [20–22].

Dipeptidyl peptidase-4 (DPP-4) inhibitors are another group of medications used for glycaemic control and treatment of DN in patients with type 2 DM [23, 24]. DDP-4 enzymes are suggested to have roles in the progression of kidney injury in patients with DN considering their inflammatory functions [25]. Therefore, inhibiting DDP-4 function is one of the therapeutic targets in patients with DN; however, there are controversies regarding the effects of DDP-4 inhibitors on kidney injury in these patients [25]. Linagliptin is one of the DDP-4 inhibitors which has been utilized for the treatment of DN. In a study by Groop et al., 5 mg of Linagliptin per day in combination with a renin–angiotensin–aldosterone system (RAAS) inhibitor had positive impacts on albuminuria and Hemoglobin A1C (HbA1C) levels in patients with type 2 DM compared to RAAS inhibitor alone [26]. However, Linagliptin did not have similar efficacy on the renal function when used alone [27].

To summarize, Empagliflozin and Linagliptin are two medications of different classes that have been used mainly for glycaemic control in patients with type 2 DM. Besides, Empagliflozin is one of the agents that has beneficial effects on renal function and has been used for this purpose. However, studies on the efficacy of Linagliptin are limited, and there are controversies in this regard. Therefore, to address these controversies on Linagliptin effects, this study aimed to compare the effects of Linagliptin and Empagliflozin on renal function and glycaemic control in patients with type 2 DM.

## Methods

We ran a randomized, double-blind, parallel trial (Iranian Registry of Clinical Trials identifier: IRCT20200722048176N1) to compare the effects of Emplagiflozin 10 mg once daily and Linagliptin 5 mg once daily on albuminuria, fasting blood sugar (FBS), HbA1c, and eGFR in patients with type 2 DM. All patients were informed in detail about the study, and verbal and written informed consent were obtained. The study design entirely was approved by the Human Ethics Committee of the Arak University of Medical Sciences (ethics code: 1399.127.REC.ARAKMU.IR).

### Trial population

The current study was conducted on patients with type 2 diabetes mellitus in Amir-al-Momenin hospital, Arak city, Iran, from September 2020, to May 2021. Patients aged 30 to 80 years and HbA1c ≤ 9% regardless of any background anti-diabetic therapy were eligible for inclusion. The dose of the background glucose-lowering drug was required to be unchanged at least 12 weeks before randomization. Exclusion criteria were: history of myocardial infarction or congestive heart failure less than three months before the study, hematuria, presence of urinary foley catheter, active urinary tract infection, and any renal diseases resulting in albuminuria at the time of inclusion.

### Intervention

Patients were divided into two groups. One group received Empagliflozin 10 mg once daily, and the other group received Linagliptin 5 mg once daily. Both medications were added to the patients’ previous anti-glycemic agents, and patients in both groups received these medications for 12 weeks. The dosage of medications was determined based on the recommendations for the treatment of patients with DM [28, 29].

### Primary and secondary outcomes

In our study, primary outcomes were changes in albuminuria and eGFR, and secondary outcomes were changes in HbA1c and FBS after 12 weeks of treatment. The subjects were required to fast for 8 to 10 h before collecting the blood samples to evaluate FBS, creatinin for calculation of GFR, and HbA1C. Also, we used a spot morning urine sample for evaluation of albuminuria. The following formula was used for the calculation of GFR based on the creatinine levels. The calculated value was multiplied by 0.85 if the patient was female.:

$$GFR\, = \,(140\, - \,age)*Weight\,(kg)/Cr\,(mg/dl)*72$$

### Blinding and randomization

For the purpose of blinding, drugs were removed from their original packages and were re-packed in look-alike packages, and neither the patients nor the instructors were informed about the contents of the packages. Patients were randomized 1:1 in a block size of 6 to receive either Empagliflozin 10 mg once daily or Linagliptin 5 mg once daily, and treatment allocation was performed by a random sequence generated by a computer. However, no allocation concealment was done.

### Statistical Analysis

We calculated the mean and standard deviation (SD) for continuous variables. We used Kolmogorov–Smirnov (KS) test to evaluate the distribution of values of each variable. We used non-parametric tests, including the Mann–Whitney U test and Wilcoxon signed ranks test, to compare the FBS, HbA1C, and albuminuria between groups as they were not distributed normally (*P* < 0.05). For other variables, we used parametric tests, such as the T-test, as they were distributed normally (*P* > 0.05). We used univariate analysis of variances to assess the effects of confounders. We calculated Cohen’s D to evaluate the effects of medications where we find significant differences in the changes of variables between groups. We considered *P* ≤ 0.05 as statistically significant. We used IBM™SPSS® statistics software for windows version 24 for statistical analysis of the data.

## Results

**Fig. 1:**
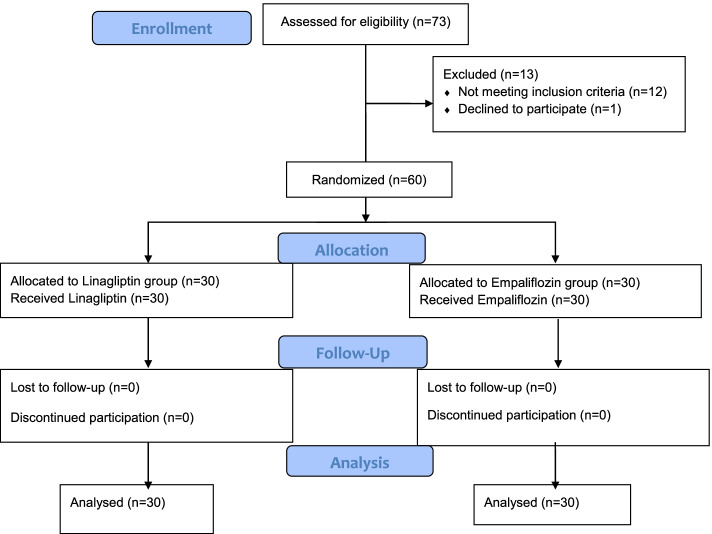
Flow diagram of participants enrollment

In total, 73 patients were assessed for the eligibility criteria, and 12 individuals were excluded as they did not meet the eligibility criteria, and one declined to participate in the study. Therefore, 60 patients were randomized into two intervention groups (Fig. 1). In both groups, nine patients were male, and 21 were female. The basic characteristics of participants are shown in Table 1. Patients who received Empagliflozin had significantly higher body mass index (BMI) than those in the Linagliptin roup (*P* = 0.049), but there were no other differences between groups regarding the basic characteristics, including age and gender (*P* > 0.05).

**Table 1:** Basic characteristics of participants

		Empagliflozin group	Linagliptin group
Gender, number (%)	Male	9 (30%)	9 (30%)
	Female	21 (70%)	21 (70%)
Age (year), mean(SD)		56.8 (8.15)	60.9 (7.22)
BMI (kg/m^2^), mean (SD)		30.12 (3.08)	28.39 (3.57)

*BMI* Body Mass Index

Clinical characteristics of participants are shown in Table 2. Before the intervention, FBS, HbA1C, and albuminuria values were significantly higher in the Empagliflozin group than those in the Linagliptin group (*P* < 0.05). After the intervention, no significant differences were seen between groups regarding the outcomes (*P* > 0.05).

**Table 2:** Primary and secondary outcomes before and after the intervention

	Pre-intervention			Post-intervention		
	Empagliflozin	Linagliptin	*P*-value	Empagliflozin	Linagliptin	*P*-value
Albuminuria (mg/24 h)	141.9 (171.76)	67.28 (38.8)	0.03	53(84.57)	66.4 (67.72)	0.739
eGFR (mL/min)	74.9 (17.05)	69.76 (18.7)	0.271	76.13 (15.95)	69.18 (17.56)	0.114
FBS (mg/dL)	184.4 (52.31)	148.9 (43.3)	0.006	143.33 (56.8)	134.16 (57.53)	0.46
HbA1C (mmol/mol)	8.99 (1.42)	8.14 (0.9)	0.015	8.18 (1.39)	7.66 (1.57)	0.09

Values are reported as mean (SD)

*FBS* Fasting Blood Suger, *eGFR* Estimated Glomerular Filtration Rate, *HbA1C* Hemoglobin A1C

GFR, albuminuria, FBS, and HbA1C values were significantly improved in patients who received Empagliflozin (*P* < 0.001). In the Linagliptin group, GFR was significantly decreased after the intervention (*P* < 0.001). Also, FBS and albuminuria did not significantly change after the treatment (*P* > 0.05). The only significant improvement in the Linagliptin group was in the HbA1C values, as HbA1C values were significantly decreased after the course of treatment (*P* = 0.002).

The decrease in albuminuria was greater in the Empagliflozin group compared to the Linagliptin group (*P* = 0.001, Cohen’s d = 0.98).). After adjusting for baseline values of albuminuria and HbA1C, changes in the albuminuria were significantly different between groups in favor of the Empagliflozin group (*P* < 0.001). Changes in GFR, FBS, and HbA1C were not significantly different between groups (*P* > 0.05).

## Discussion

In this double-blind, randomized clinical trial comparing the effectiveness of Empagliflozin and Linagliptin in patients with type 2 DM, Empagliflozin emerged superior in efficacy regarding reducing albuminuria in the short term follow up of 12 weeks, regardless of their pre interventional renal function, BMI, and HbA1C, which is in line with previous studies. In a study by Lee et al., comparing the adverse kidney outcomes in patients with type 2 DM who received Empagliflozin or Linagliptin, it was found that decline in GFR was slower in patients who received Empagliflozin compared to those who received Linagliptin [30]. Also, they found that the risk of acute kidney injury (AKI) is lower in patients who receive Empagliflozin compared to those who received Linagliptin [30]. These findings indicate that Empagliflozin might be superior than Linagliptin in the management of DN not only in the trial condition, but also in the real-world situation.

In 2017 Groop et al. conducted a randomized clinical trial, MARLINA, comparing the effectiveness of Linagliptin versus placebo in reducing the albuminuria in patients with type 2 DM in a more extended period of follow up, 24 weeks [31]. In their study, no superiority was found in favor of Linagliptin in reducing albuminuria. However, in another randomized trial, CARMELINA, after a median follow-up of 2.2 years, Linagliptin reduced the albuminuria compared to placebo [32]. Although it has been reported that Linagliptin can prevent the progression of albuminuria in 2.2 years in different studies [33, 34], its efficacy in reducing albuminuria in the short term is still controversial. In a retrospective analysis of four randomized trials, significant efficacy of Linagliptin in lowering the albuminuria 24 weeks after randomization was found [26]. Han et al. in 2017 enrolled patients with an estimated eGFR of 15–59 in a randomized clinical trial to compare the efficacy of Linagliptin with Empagliflozin. After 40 weeks: they observed no statistically significant change in albuminuria in either group in this period [35].

Glycoprotein DPP-4, which has been found in healthy individuals in two forms of circulating soluble and membrane-bound with a predominancy in proximal convoluted tubules [36], was also expressed in glomeruli as an adaptive mechanism in patients with chronic kidney disease [37]. So a hypothesis was brought out of the renoprotective role of DPP-4 inhibitors and later demonstrated a reduction in albuminuria in mice with type 2 DM [38]. As a DPP-4 inhibitor, Linagliptin has shown to act as a renoprotective agent through the prevention of endothelial to mesangial transition [39] and its anti-fibrotic effects [40, 41]. For example, in an experimental study in 2016 in mice, using Linagliptin, a DPP-4 inhibitor, was associated with upregulation of stromal cell-derived Factor-1, which contributes as an antioxidative and anti-fibrotic agent in the pathophysiology of diabetic nephropathy [42]. Another study investigating the role of DPP-4 in kidney disease suggested Linagliptin as an inhibitor of podocyte growth, which could reduce albuminuria with Linagliptin in the long term [43].

On the other hand, SGLT2, a transport protein in proximal convoluted tubules contributing to sodium-glucose reabsorption, can be inhibited by Empagliflozin, mediating the reduction in blood pressure intraglomerular filtration, which seems to lead to a reduction in albuminuria in a short time. Beyond alleviation of patients’ hemodynamic status, histopathologic effects of SGLT-2 inhibitors could mediate a reduction in their albuminuria. In 2020, Klimontov et al. administered Empagliflozin to diabetic mice. As well as a reduction of urinary albumin-to-creatinine ratio, it showed a reduction in kidney hypertrophy, mesangial expansion, basement membrane thickening, and podocytopathy of glomeruli [44] that could explain its long-term efficacy in patients with DN.

Beyond Empagliflozin association with eGFR preservation and reduced risk of major adverse kidney events reported in a cohort study of 379,033 participants [45] and EMPA-REG trial [46], several studies have been designed to evaluate its efficacy in reducing albuminuria. Cherney et al. in 2017 evaluated the efficacy of Empagliflozin compared to placebo in reducing albuminuria in a median treatment duration of 2.6 years, and in line with the current study, they found the reduction in UACR occurs as early as week 12 [47]. Furthermore, a post hoc analysis of the EMPA-REG OUTCOME trial revealed a sustained reduction in UACR after a median follow-up of 3.1 years [48].

An intriguing conclusion was brought out of the EMPA-REG trial regarding the importance of early albuminuria reduction, where each 30% reduction of UACR was associated significantly with a lower hazard for major cardiovascular events [49]. In this regard, decreasing albuminuria in diabetic patients as early as possible could be very beneficial. To the best of our knowledge, this current study is the first clinical double-blind trial that revealed the superiority of Empagliflozin to Linagliptin. In a randomized trial by Cooper et al. in 2018, it was shown that Linagliptin did not change the hemodynamic status of patients with Type 2 DM in a period of 24 weeks [50], while some other studies, including a clinical trial of patients with Type 2 DM [51] and a post hoc analysis of two cohorts of randomized trials in patients with Type 2 DM [52], showed the Empagliflozin reducing effect on both systolic and diastolic blood pressure at weeks 8, 12, and 24, respectively.

There was a heterogeneity regarding the FBS, HbA1C, and albuminuria values between the groups, which should be considered in the interpretation of the current study’s findings. We used blocked randomization in our study to ensure the same number of participants would be enrolled in each group. However, there is a risk of imbalance regarding the prognostic factors while using block randomization, which might be even increased in the context of our study considering the small number of our sample size [53]. Adjusting for baseline values of albuminuria and HbA1C before comparing changes in the albuminuria between groups was a strategy used in this study to overcome this limitation. Also, baseline characteristics of participants indicated poorer diabetes control in individuals in the Empagliflozin group, which might be due to less adherence to treatment or being in more advanced stages of diabetes. Therefore, observing the effectiveness of Empagliflozin compared to Linagliptin even in patients with poorer conditions might be another factor in favor of Empagliflozin. Also, we did not perform allocation concealment due to limitations in resources for conducting this study in our center, which is another factor that should be considered in the interpretation of the findings as due to lack of allocation concealment, our study might be subject to selection bias [54].

This study has several limitations suggested to be considered in future studies. First of all, it is better to follow up patients for more extended periods to meet the albuminuria-lowering effects of Linagliptin and their probable adverse complications. On the other hand, stratified randomized sampling according to potential confounders would yield more reliable findings, and a larger sample size with various ethnicities could enhance the power of the study. Also, we did not record the anti-glycemic agents that patients were taking before participating in this study, which can be a confounder for our results. Future studies evaluating pre-enrollment medications may be useful for more careful comparison of the effects of these medications. Finally, we did not perform allocation concealment and did not record the side effects in this study, and future well-designed controlled trials are needed to compare the effects of Empagliflozin and Linagliptin better.

## Conclusion

Regardless of baseline albuminuria, eGFR, or HbA1c, Empagliflozin 10 mg daily significantly reduced albuminuria at 12 weeks compared to Linagliptin 5 mg daily in patients with type 2 diabetes.

## Data Availability

The datasets generated during and/or analyzed during the current study are available from the corresponding author on reasonable request.

## Abbreviations

BMI: Body Mass Index
DM: Diabetes Mellitus
DN: Diabetic Nephropathy
DPP-4: Dipeptidyl Peptidase-4
eGFR: Estimated Glomerular Filtration Rate
ESRD: End-Stage Renal Disease
FBS: Fasting Blood Sugar
HbA1C: Hemoglobin A1C
KS: Kolmogorov–Smirnov
RAAS: Renin–Angiotensin–Aldosterone System
RAS: Renin-Angiotensin System
ROS: Reactive Oxygen Species
SD: Standard Deviation
SGLT2i: Sodium-Glucose Cotransporter-2 inhibitors
